# Adolescent girls’ health, nutrition and wellbeing in rural eastern India: a descriptive, cross-sectional community-based study

**DOI:** 10.1186/s12889-019-7053-1

**Published:** 2019-05-31

**Authors:** Kelly Rose-Clarke, Hemanta Pradhan, Suchitra Rath, Shibanand Rath, Subhashree Samal, Sumitra Gagrai, Nirmala Nair, Prasanta Tripathy, Audrey Prost

**Affiliations:** 1 Department of Global Health and Social Medicine, King’s College London, Bush House NE Wing, London, WC2B 4BG UK; 2 Ekjut, Chakradharpur, Jharkhand India; 3 Institute for Global Health, University College London, London, UK

**Keywords:** Adolescent health, Mental health, Undernutrition, Violence, Sexual and reproductive health, Education, Menstrual hygiene, India

## Abstract

**Background:**

India is home to 243 million adolescents. Two million (9%) of them belong to Scheduled Tribes living in underserved, rural areas. Few studies have examined the health of tribal adolescents. We conducted a cross-sectional survey to assess the health, nutrition and wellbeing of adolescent girls in rural Jharkhand, eastern India, a state where 26% of the population is from Scheduled Tribes. We aimed to identify priorities for community interventions to serve adolescents and their families.

**Methods:**

Between June 2016 and January 2017, interviewers visited all households in 50 purposively sampled villages of West Singhbhum district, Jharkhand. They aimed to interview all girls aged 10–19. Interviewers conducted face-to-face interviews with girls to administer a survey about physical and mental health, disability, nutrition, sexual and reproductive health, gender norms, decision-making, education and violence. Interviewers also measured girls’ height, weight, and Mid-Upper Arm Circumference.

**Results:**

Interviewers collected data from 3324 (82%) of an estimated 4068 girls residing in the study area. Their mean age was 14.3 (SD 2.9). 82% were from Scheduled Tribes. 89% of younger girls aged 10–14 and 46% of older girls aged 15–19 were in school or college. Girls dropped out of school because they were required for household work (37%) or work on the family farm or business (22%). Over a third reported symptoms of anaemia in the past month, but less than a fifth had a blood test. The prevalence of thinness (<−2SD median BMI for age and sex) was 14% for younger girls and 6% for older girls. 45% of girls were stunted (<−2SD median height for age and sex). 40% reported emotional violence in the past year, 14% physical violence, and 0.7% sexual violence. 12% had problems associated with depression or anxiety. 30% aged 15–19 had heard of contraception. Among married girls and their husbands, only 10% had ever used methods to prevent or delay pregnancy.

**Conclusions:**

Our study identified several priorities to improve adolescent girls’ health, nutrition and wellbeing in largely tribal areas of Jharkhand: reducing violence, early marriage and undernutrition, as well as improving mental health, knowledge about contraception and school retention.

**Electronic supplementary material:**

The online version of this article (10.1186/s12889-019-7053-1) contains supplementary material, which is available to authorized users.

## Background

Global investment in adolescent health is crucial. Adolescents aged 10–19 years constitute around one sixth of the world’s population, account for 6% of the global burden of disease and injury, and suffer over 1.2 million deaths each year [1]. While infant (first year of life) and childhood (1–9 years) mortality have declined substantially in the last 50 years, reductions in adolescent mortality have been more modest [2]. Poor health or risky behaviours during adolescence can have negative impacts on health in adult life [3]. In addition, childbearing in adolescence can have adverse effects across generations by increasing the risk of low birth weight and poor growth, and by perpetuating the intergenerational cycle of poverty [4]. For these reasons, the Global Strategy to Improve Women’s, Children’s and Adolescents’ Health (2016–2030) includes a commitment to accelerate action to improve adolescent health [5].

India has the largest cohort of adolescents in the world, approximately 243 million [6]. A recent national review found that adolescents were commonly affected by both under and overnutrition, common mental disorders, substance use and violence [7]. Girls are particularly vulnerable: 45% of girls aged 15–18 have a BMI less than 18.5, and 27% of women aged 20–24 were married before the age of 18 [8, 9]. Sixty-eight percent of women are literate compared to 86% of men [8]. These national figures mask important inequities across States, and between wealth and caste/tribal groups. Scheduled Tribe, or *adivasi* (i.e. indigenous) communities constitute 9% of India’s population - over 104 million people [10]. Although concentrated in Central, Eastern and North East India, such communities exist across India. They have historically been socio-economically disadvantaged, disproportionately affected by undernutrition, and underserved by health services [11]. Despite these inequities, there is little recent data on the health, nutrition and wellbeing of adolescents in Scheduled Tribe communities.

We aimed to assess the health, nutrition and wellbeing of adolescent girls aged 10–19 years living in a predominantly tribal area of rural Jharkhand, India. We sought to identify priorities for community intervention at the State level, with broader implications for tribal adolescent health across the country.

## Methods

### Study design

Between June 2016 and January 2017, we conducted a cross-sectional, community-based survey of adolescent girls in West Singhbhum district, Jharkhand, a state of eastern India. This study was the baseline survey to inform the design of a cluster-randomised controlled trial (ISRCTN17206016) of an intervention to improve adolescent health with Ekjut, a civil society organisation based in Jharkhand, in partnership with University College London.

### Setting

The size of the study area was chosen for logistical reasons and comprised 50 villages and their attached hamlets in Khuntpani block, West Singhbhum district, southern Jharkhand. Over a quarter of Jharkhand’s population is from Scheduled Tribes [10]. The majority of the population living in our study villages were from the Ho tribe, a tribal group comprising over 1 million people, around 930,000 of whom reside in Jharkhand [12]. The Ho families in our study areas were mainly engaged in cultivation and seasonal migration for wage labour.

Because this study was the baseline survey for a cluster randomised controlled trial, the 50 villages and hamlets were grouped into 38 population clusters. Each cluster was a purposively selected geographic area with a population of around 1000 (range: 723–1962). All of the 50 study villages had an Anganwadi centre providing basic healthcare for mothers and children. Eleven out of 50 had a sub-centre usually staffed by an Auxiliary Nurse Midwife (female) and a multipurpose worker (male). Most villages (33/50) had a primary school and 19 had a secondary school.

### Survey methods

#### Inclusion criteria

We aimed to interview all married and unmarried adolescent girls aged 10–19 living in the study area.

#### Recruitment process

An interviewer visited each household outside school hours to identify eligible girls, obtain their consent for participation as well as the consent of their caregiver, and to conduct face-to-face interviews with girls. All interviewers were female. Depending on the participant’s preference, interviews were conducted in Hindi, Ho or Oriya by interviewers who were fluent in these languages. If an eligible girl was unavailable due to e.g. attending a boarding school or living in a hostel, the reason was documented. Interviewers did not make a repeat visit to the household.

#### Survey instrument development

Our survey instrument is provided as an additional file (Additional file 1). The instrument included questions on physical and mental health, disability, nutrition, sexual and reproductive health, gender norms, decision-making, education and violence. Interviewers also conducted short interviews with girls’ caregivers (or girls themselves if aged 18 or older) to collect household-level socioeconomic data.

For some areas of adolescent health, we were able to adopt questions used in previous national surveys of adolescents in India and adolescent health indicators from the international literature [13–15]. For example, we assessed literacy by asking girls to read a standard sentence, as in India’s National Family Health Survey. We asked about disability using the Washington Group Short Set of Disability Questions [16]. These assess whether participants have difficulty with walking, seeing, hearing, cognition, self-care or communication, are suitable for children aged 5 years and above, and have been used extensively in India [17].

Similarly, to assess girls’ nutritional status, we used standard international indicators and methods. We measured girls’ height using a Seca 213 stadiometer, their weight using a Seca 874 scale, and Mid Upper Arm Circumference (MUAC) using a standard adult tape procured from UNICEF. We defined stunting (height for age) and thinness (BMI for age) as − 2 Standard Deviations (SD) below the mean sex-specific WHO Reference 2007 [18]. We defined overweight as + 2 SD above the mean. In reporting BMI-for-age statistics, we excluded girls who were pregnant for the first time, and girls who had already been pregnant if it was unclear whether or not they were pregnant at the time of the survey. We calculated the proportion of girls aged 15–19 with a BMI less than 18.5. There is no standardised MUAC cut-off to identify adolescent thinness, so we used a cut-off of < 160 mm among girls aged 10–14 years based on nutrition guidelines for HIV-infected children [19]. Interviewers received anthropometry training and participated in a standardisation exercise with 10 adolescent girls to assess Technical Error of Measurement. The inter-observer coefficient of reliability was 0.98 for height and 0.99 for weight and MUAC. Each measurement was repeated and we calculated a mean of the two measurements. We used the Food and Nutrition Technical Assistance (FANTA) tool to measure minimum dietary diversity, i.e. the proportion of girls who had consumed five or more of 10 key food groups in the last day or night [20].

We assessed knowledge and practices related to sexual and reproductive health among all girls aged 15–19 (married and unmarried) as well as girls aged 10–14 who were married. We used questions from the Youth in India: Situation and Needs Study 2005–6 [14]. We asked girls whether they had heard about contraception, what kind, from where, and about whether they thought abortion was legal. We also asked about previous pregnancies. Married girls were asked whether they or their husband had used any methods to prevent or delay pregnancies.

For other areas of adolescent health however, there were no validated instruments or internationally recognised indicators. For example, there are no appropriate brief screening tools for adolescent mental health validated in India. We decided to use the Brief Problem Checklist (BPC), which comprises a six-item Internalising scale and a six-item Externalising scale, as well as a Total Problems scale. This tool was developed and validated for a US clinical population and was shown to be both easily administered and psychometrically strong [21]. Interviewers in our study found the BPC simple to use and participants understood the items easily. In our sample, Cronbach’s alpha, a measure of internal consistency, was 0.77. The BPC has now been replaced by the Brief Problem Monitor (BPM) [22].

Collecting data on violence was equally challenging. We asked about girls’ exposure to emotional, physical and sexual violence using a translated and modified version of the Child Abuse and Neglect Screening Tool – Child Version (ICAST-C) [23]. The tool was adapted to shorten administration and to reflect local forms of violence such as being made to work or look after siblings, and exposure to ‘societal violence’ defined as witch hunting, communal violence, social boycott, being prevented from accessing public facilities or common resources, or being subjected to a community-imposed penalty.

### Data management and statistical methods

We collected data using smartphones programmed with CommCare software [24]. We coded and analysed data in Stata version 14 [25]. For nutrition data, we computed height for age and BMI for age z-scores with a Stata macro for the WHO Reference 2007.

We report descriptive statistics (mean, SD, range) for each area of adolescent health need. We present results for younger (10–14 years) and older (15–19 years) adolescents as well as for the total sample because these age groups differ and there is relatively little information on the younger age group in particular.

### Ethical considerations

The study was approved by an independent ethics board convened by Ekjut in Ranchi, Jharkhand, and by the Research Ethics Committee of University College London. Ekjut had been working in the study districts and collecting data on maternal and child health in tribal communities and with local data collectors for 10 years, so had a good rapport with the study communities. However, the fact that the study involved asking adolescent girls questions about potentially sensitive issues presented substantial ethical challenges.

#### Consent

We sought consent for each village’s participation from the local village governance institutions (Panchayat and headmen) and opinion leaders after explaining the study’s purpose and processes. Interviewers explained to all adolescents and any parents or husbands that the survey would include questions on health, nutrition, as well as potentially more sensitive subjects such as alcohol and tobacco use, feelings and worries about growing up, about safety, or about experiences at school and at home. Interviewers said that participation was voluntary, that choosing not to participate would not disadvantage the family or adolescent in any way, and that participants could stop the interview at any time or skip any questions that they did not want to answer. We obtained consent (a witnessed thumbprint) from all girls who participated in the study. For girls younger than 18 years, we also sought consent from their caregiver. For married adolescent girls younger than 18 years, we sought consent from the husband, though married adolescent girls were interviewed privately. We did not collect data from any girls if they had not themselves provided informed consent, regardless of whether or not their caregiver or husband had.

#### Confidentiality

Data collectors needed to be able to speak Hindi and Ho, but could not be from the villages in which interviews were being conducted in order to protect participants’ confidentiality. We followed WHO ethical and safety recommendations for research on domestic violence against women by always asking questions about violence in a private space, with no third-party present, and within the bounds of a more general survey [26]. All data collected on smartphones were anonymised following downloads, and interviewers had password protected smartphones.

#### Referrals

Ekjut convened a local multidisciplinary child protection committee to help coordinate the referral of vulnerable adolescents identified through the research. The committee included the Child Protection Officer and Probation Officer from the District Child Protection Unit, all members and the Chairperson from the Child Welfare Committee, the Office Legal Assistant from the District Legal Services Authority, and NGO representatives. We used the data collection software (CommCare) to flag participants who had faced sexual or physical violence, as well as those with severe mental health problems or severe undernutrition to each interviewer. The interviewers and their supervisors then confidentially offered help from a trained psychosocial counsellor to visit these adolescents, discuss their needs and organise any onward referral with explicit consent from the adolescent.

## Results

We estimated that 4068 girls aged 10–19 lived in our 50 study villages based on projections using data from the 2011 Indian Census [27]. In total, we identified 3932 (97%) girls and interviewed 3324 (82%). The most common reasons for being unavailable for interview included boarding at a hostel away from home in order to study or work, and being married and living in the marital home.

### Sample characteristics

Girls had a mean age of 14.3 (SD 2.9) and 54% were aged 10–14 (Table 1). Most (82%) were from Scheduled Tribes, mainly the Ho tribe, or from Other Backward Classes (17.4%). A fifth of girls aged 15–19 were married compared to 0.3% of girls aged 10–14. The majority (85%) lived in households with electricity (85%) but 92% did not have access to a toilet.

**Table 1:** Socio-economic characteristics of adolescent girls in the study area

	10–14 years		15–19 years		Total (*n* = 3324)	
	N	%	N	%	N	%
Household location						
Main Village	1297	72.8	1077	69.8	2374	71.4
Hamlet	485	27.2	465	30.2	950	28.6
Class/Caste status						
Scheduled Tribe	1467	82.3	1264	82.0	2731	82.2
Scheduled Caste	9	0.5	6	0.4	15	0.5
Other Backward Class	306	17.2	272	17.6	578	17.4
Tribe (*n* = 2731)						
Ho	1449	98.8	1254	99.2	2703	99.0
Santhal	8	0.5	2	0.2	10	0.4
Oraon	0	0.0	1	0.1	1	0.0
Munda	10	0.7	6	0.5	16	0.6
Other	0	0.0	1	0.1	1	0.0
Religion						
Sarna	1463	82.1	1250	81.1	2713	81.6
Hindu	247	13.9	222	14.4	469	14.1
Christian	47	2.6	52	3.4	99	3.0
Other	25	1.4	18	1.2	43	1.3
Marital status						
Not married	1777	99.7	1196	77.6	2973	89.4
Married	5	0.3	346	22.4	351	10.6
Owns personal mobile phone	37	2.1	466	30.2	503	15.1
Household assets^a^						
Bicycle	1532	86.0	1288	83.5	2820	84.8
Television	151	8.5	147	9.5	298	9.0
Electricity	1503	84.3	1325	85.9	2828	85.1
Motorcycle	203	11.4	185	12	388	11.7
Sources of household income^a^						
Daily paid labour through MGNREGA^b^	745	41.8	653	42.3	1398	42.1
Daily paid labour not through MNREGA	1411	79.2	1276	82.7	2687	80.8
Paid agricultural labour	1163	65.3	991	64.3	2154	64.8
Small scale trade (tela, small market stall, very small shop)	106	5.9	74	4.8	180	5.4
Type of toilet in household						
Not improved	41	2.3	49	3.2	90	2.7
Improved	111	6.2	76	4.9	187	5.6
Field	1630	91.5	1417	91.9	3047	91.7

^a^ Participants selected one or more options as appropriate; ^b^MGNREGA Mahatma Gandhi National Rural Employment Guarantee Act, a national programme to provide wage employment to households in rural area

### Education

**Fig. 1:**
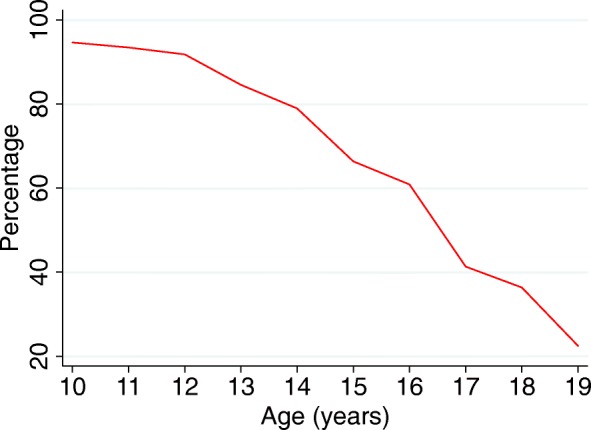
School or college attendance by age among adolescent girls in Jharkhand

Twenty-three percent of older girls aged 15–19 were unable to read and 13.5% had never been to school (Table 2). Fewer younger girls aged 10–14 were illiterate (14.6%) and 3.7% had never attended school. The proportion of girls attending school or college reduced with age, particularly from 12 years (Fig. 1). The most common reasons for never attending school were being required for household work (53%), being required for work on the family farm or in the family business (38%), prohibitive school costs (24%) and views that education was not necessary (20%). The most common reasons for dropping out of school were linked to being required for household work, work on the family farm or in the family business. Fourty-4 % of girls had been absent from school or college in the past 2 weeks, mainly due to household work, work on the family farm or business, or personal illness. Only 1% of girls had received any vocational training in the past year.

**Table 2:** Educational status of adolescent girls in the study area

	10–14 years		15–19 years		Total	
	N	%	N	%	N	%
Literacy						
Cannot read	260	14.6	347	22.5	607	18.3
Reads with difficulty	677	38.0	311	20.2	988	29.7
Reads easily	845	47.4	884	57.3	1729	52.0
School attendance						
Never attended school	65	3.7	208	13.5	273	8.2
Previously attended school	136	7.6	624	40.5	760	22.9
Currently attends school or college	1581	88.7	710	46.0	2291	68.9
Reasons for never attending school (*n* = 273) ^a^						
Education not considered necessary	9	13.8	47	22.6	56	20.5
Required for work on the family farm or in the family business	18	27.7	87	41.8	105	38.5
Costs too much money	12	18.5	54	26.0	66	24.2
Required for household work	35	53.8	110	52.9	145	53.1
Reasons for dropping out of school (*n* = 760) ^a^						
Required for household work	57	41.9	228	36.5	285	37.5
Required for work on the family farm or in the family business	31	22.8	140	22.4	171	22.5
Failed exams	6	4.4	131	21.0	137	18.0
Illness or death of family member	29	21.3	99	15.9	128	16.8
Got married	2	1.5	114	18.3	116	15.3
Absence in the past 2 weeks (*n* = 2267)	725	46.0	274	39.6	999	44.1
Reason for absence (*n* = 999)						
Festival	106	14.6	31	11.3	137	13.7
Strikes/bandhs	13	1.8	7	2.6	20	2.0
Required to work on family farm or in the family business	151	20.8	76	27.7	227	22.7
Required for household work	195	26.9	69	25.2	264	26.4
Death or illness of a family member	28	3.9	9	3.3	37	3.7
Personal illness	139	19.2	33	12.0	172	17.2
No reason	43	5.9	17	6.2	60	6.0
Other	50	6.9	32	11.7	82	8.2
Received vocational training in the last year	12	0.7	33	2.1	45	1.4

^a^ Participants selected one or more options as appropriate

### General health and nutrition

The prevalence of disability was 0.4%. Most girls reported that they felt ‘quite healthy’ (77%) or ‘very healthy’ (13%) (Table 3). Common health problems reported by girls included high fever (19%), menstrual problems (16%) and vomiting or nausea (8%). Less than half of those who had problems said they sought help for these. Among those who did seek help, they mainly consulted informal and largely untrained allopathic providers referred to as ‘village doctors’ (27%), pharmacist (10%) or traditional healers locally termed ‘Ojha’. Fourteen percent of younger girls and 15% of older girls had used alcohol in the past month.

**Table 3:** General health, nutrition and menstrual hygiene among adolescent girls in the study area

	10–14 years		15–19 years		Total	
	N	%	N	%	N	%
GENERAL HEALTH						
Self-rated health						
Very Healthy	206	11.6	220	14.3	426	12.8
Quite Healthy	1410	79.1	1141	74.0	2551	76.7
Not Very Healthy	166	9.3	181	11.7	347	10.4
Disability	10	0.6	4	0.3	14	0.4
Health problem ^a^						
High fever	330	18.5	301	19.5	631	19.0
Vaginal discharge	105	5.9	154	10	259	7.8
Menstrual problems	123	6.9	397	25.7	520	15.6
Diarrhoea	35	2.0	47	3.0	82	2.5
Vomiting or nausea	140	7.9	137	8.9	277	8.3
Sought help for health problems (*n* = 1269)	245	41.7	286	41.9	531	41.8
Sources of help for health problems (*n* = 1269) ^a^						
Pharmacist	56	9.5	70	10.3	126	9.9
Village doctor	161	27.4	178	26.1	339	26.7
Traditional healer (Ojha)	22	3.7	62	9.1	84	6.6
Used tobacco in past month	7	0.4	22	1.4	29	0.9
Drank alcohol in past month	245	13.7	230	14.9	475	14.3
NUTRITION						
Thinness (BMI < -2SD) (*n* = 3275)	254	14.3	96	6.4	350	10.7
Overweight (BMI > 1SD) (*n* = 3275)	34	1.9	16	1.1	50	1.5
BMI < 18.5 (*n* = 1493)	n/a	n/a	609	40.8	n/a	n/a
Stunting (height < −2SD) (*n* = 3323)	590	33.1	898	58.2	1488	44.8
Mean MUAC (SD)	19.8	2.5	23.2	2.0	21.4	2.8
MUAC < 160 mm	64	3.6	n/a	n/a	n/a	n/a
Minimum dietary diversity	425	23.8	331	21.5	756	22.7
Symptoms of anaemia in past month^b^	560	31.8	688	44.8	1248	37.8
Blood test for symptoms of anaemia in past month (*n* = 1248)	102	18.2	145	21.1	247	19.8
MENSTRUAL HYGIENE						
Started period	698	39.2	1522	98.7	2220	66.8
Mean age started period (mean, SD)	12	0.9	12.3	1.0	12.2	1.0
Method of sanitary protection (*n* = 2220)						
Nothing	45	6.4	76	5.0	121	5.5
Any cloth	118	16.9	248	16.3	366	16.5
Locally made napkins	177	25.4	433	28.4	610	27.5
Sanitary napkins	338	48.4	738	48.5	1076	48.5
Other method	20	2.9	27	1.8	47	2.1
Restriction during menstruation (*n* = 2220)^a^						
Restricted participation in religious rituals	30	4.3	75	4.9	105	4.7
Restricted participation in sports	8	1.1	14	0.9	22	1.0
Restricted foods	12	1.7	17	1.1	29	1.3
Restricted access to the kitchen	11	1.6	26	1.7	37	1.7

^**a**^ Participants selected one or more options as appropriate; ^**b**^ Weakness, tired all the time, pale hands and face, dizziness, breathlessness when resting

The prevalence of thinness (<−2SD BMI for age and sex) among younger girls (14%) was more than twice the prevalence among older girls (6%). However, 41% of older girls had a BMI below 18.5. Only 1% of girls aged 10–19 were overweight (BMI >1SD). Stunting (<−2SD height for age and sex) was common, affecting a third of younger girls and 58% of older girls. Less than a quarter of all girls achieved minimum dietary diversity in the previous 24 h. The mean MUAC measurement for younger girls was 19.8 cm and 23.2 cm for older girls. Although 38% of girls reported experiencing symptoms of anaemia (weakness, tired all the time, pale hands and face, dizziness, breathlessness when resting) only a fifth had a blood test for these symptoms.

The mean age of menarche was 12 years (SD 1.0). Half the girls used sanitary napkins and 27% used locally made napkins. Restrictions during menstruation were uncommon and mainly pertained to participation in religious rituals.

### Sexual and reproductive health

We asked all girls aged 15–19 and any married girls aged 10–14 (*n* = 1547) questions related to sexual and reproductive health (Table 4). Only 30% of girls had heard of contraception. Among those who had heard of contraception, 92% had heard of oral contraceptive pills and 22% had heard of condoms. A quarter of girls who had heard of contraceptive pills knew that they should be taken every day. Of those who had heard of condoms, 45% knew they should be used for only one act of sexual intercourse. Thirty-eight percent of girls who had heard of IUDs knew that they are placed in the uterus. Only 7% knew that abortion is legal in India (84% thought abortion was not legal and 9% did not know). Friends or neighbours were the most common sources of information on contraception. Nine percent of girls had been pregnant and 68% of these girls had had a pregnancy under the age of 18. Among married girls, only 10% had ever used contraception with their husband and oral contraceptive pills were the most common method used.

**Table 4:** Sexual and reproductive health knowledge and behaviours among married girls and girls aged 15–19 years in the study area

	N	%
Heard of contraception (*n* = 1547)	464	30.0
Types of contraception heard about (*n* = 464) ^a^		
Regular oral contraceptive pills	425	91.6
Injection	98	21.1
Condom	100	21.6
Female sterilisation	66	14.2
Male sterilisation	57	12.3
Emergency contraceptive pills	34	7.3
IUD	27	5.8
Withdrawal	13	2.8
Sources of information on contraception (*n* = 464) ^a^		
Teacher	128	27.6
Mother	103	22.2
Brother in-law	78	16.8
Sister	88	19
Husband	56	12.1
Other family members	206	44.4
Friends or neighbours	390	84.1
Books, newspapers or magazines	59	12.7
Television	103	22.2
Correct knowledge about oral contraceptive pills (*n* = 675)	162	24.0
Correct knowledge about condoms (*n* = 363)	162	44.6
Correct knowledge about IUDs (*n* = 182)	69	37.9
Knowledge about legality of abortion (*n* = 1547)		
Thinks abortion is legal	104	6.7
Thinks abortion is not legal	1302	84.2
Does not know or cannot say	141	9.1
Ever been pregnant (*n* = 1547)	138	8.9
Had a pregnancy before age 18 (*n* = 138)	94	68.1
First pregnancy wanted or unwanted (*n* = 138)		
Neither mother nor father wanted the pregnancy	6	4.4
Only mother wanted pregnancy	2	1.5
Only father wanted pregnancy	3	2.2
Both mother and father wanted pregnancy	127	92.0
You or your husband ever used methods to prevent or delay pregnancy (*n* = 341)	34	10.0

^**a**^ Participants selected one or more options as appropriate, *IUD* intrauterine device

### Violence and mental health

Exposure to violence in the past year was common, especially among younger girls (Table 5). Fourty-4 % of girls aged 10–14 years had been exposed to emotional violence, 18% to physical violence and 0.3% to sexual violence. Among older girls, 37% had experienced emotional violence, 9% physical violence and 1% sexual violence. Parents, siblings and other relatives were the most common perpetrators of violence for both age groups. Compared to older girls, a higher proportion of younger girls had experienced violence perpetrated by peers (unrelated children or adolescents). Over half had witnessed their parents or guardians shouting at each other and almost a quarter had seen or overheard their father hit or beat their mother.

**Table 5:** Violence exposures and mental health among girls aged 10–19 years in the study area

	10–14 years		15–19 years		Total	
	N	%	N	%	N	%
VIOLENCE						
Any form of violence in the past year (*n* = 3288)	836	47.6	569	37.2	1405	42.7
Emotional violence in past year (*n* = 3293)	767	43.5	543	35.5	1310	39.8
Physical violence in past year (*n* = 3299)	325	18.4	135	8.8	460	13.9
Sexual violence in past year (*n* = 3323)	6	0.3	17	1.1	23	0.7
Perpetrators of emotional, physical or sexual violence in last year ^a^						
Parents (*n* = 1405)	621	74.3	356	62.6	977	69.5
Sibling (*n* = 1405)	374	44.7	235	41.3	609	43.3
Husband (*n* = 1405)	2	0.2	59	10.4	61	4.3
Parent in-law (*n* = 1402)	0	0	40	7.1	40	2.9
Other relative (*n* = 1405)	293	35	227	39.9	520	37
Unrelated child/adolescent (*n* = 1405)	232	27.8	91	16	323	23
Employer (*n* = 1405)	3	0.4	4	0.7	7	0.5
Teacher (*n* = 1405)	55	6.6	14	2.5	69	4.9
Unrelated adult (*n* = 1405)	75	9	41	7.2	116	8.3
Sought medical attention as a result of violence (*n* = 597)	29	7.5	22	10.4	51	8.5
Societal violence in past year (*n* = 3084)	132	8.1	132	9.1	264	8.6
Seen or overheard parents/guardians shouting at each other (*n* = 3324)	1005	56.4	777	50.4	1782	53.6
Seen or overheard father hit or beat mother (*n* = 3324)	503	28.2	320	20.8	823	24.8
Seen or overheard mother hit or beat father (*n* = 3324)	201	11.3	87	5.6	288	8.7
MENTAL HEALTH						
Answered ‘Somewhat true’ or ‘Very true’ to all BPC items (*n* = 3131)	41	2.4	23	1.6	64	2.0
Answered ‘Somewhat true’ or ‘Very true’ to all externalising BPC items (*n* = 3131) ^b^	71	4.2	38	2.6	109	3.5
Answered ‘Somewhat true’ or ‘Very true’ to all internalising items (*n* = 3131) ^c^	207	12.3	174	12.0	381	12.2
Seriously considered suicide in past year (*n* = 3324)	66	3.7	97	6.3	163	4.9
Made a suicide plan in past year (*n* = 163)	34	51.5	60	61.9	94	57.7
No. of times attempted suicide in past year (*n* = 163)						
Never	14	21.2	16	16.5	30	18.4
1 time	21	31.8	40	41.2	61	37.4
2 or 3 times	30	45.5	27	27.8	57	35.0
4 or 5 times	0	0.0	8	8.2	8	4.9
6 or more times	1	1.5	6	6.2	7	4.3
Think parents or guardians understand problems (*n* = 3324)						
Never	192	10.8	149	9.7	341	10.3
Rarely	162	9.1	105	6.8	267	8.0
Sometimes	171	9.6	136	8.8	307	9.2
Most of the time	384	21.5	315	20.4	699	21.0
Always	873	49.0	837	54.3	1710	51.4
Has close friends (*n* = 3324)	1553	87.1	1191	77.2	2744	82.6

^**a**^ Participants selected one or more options as appropriate; ^**b**^items relate to conduct disorder and oppositional defiant disorder and enquire about tendency to argue, destroy things, disobey parents or people at school, stubbornness, anger and threatening to hurt people; ^**c**^items relate to depression and anxiety and enquire about feeling worried, unhappy, sad or depressed, fearful or anxious, self-conscious or embarrassed, worthless or inferior, and guilty

We used the BPC to assess mental health problems. 12% of girls answered ‘Somewhat true’ or ‘Very true’ to all internalising items relating to depression and anxiety (e.g. items about sadness, guilt, anxiety and worthlessness). Only 3% answered ‘Somewhat true’ or ‘Very true’ to all externalising items relating to conduct disorder and oppositional defiant disorder (e.g. items about arguing, being stubborn, having a temper). Slightly more older girls had seriously considered suicide (6%) in the past year compared to younger girls (4%). Of those who had considered suicide, more than half had made a suicide plan and most had attempted suicide at least once. Although the majority of girls reported that they had close friends, fewer older girls had close friends than younger girls.

## Discussion

We have undertaken the most recent, comprehensive survey of adolescent health among Indian tribal communities in eastern India to date. Our study provides data for both younger and older girls, addressing an important gap in the research literature, especially for girls aged 10–14. There were key differences in the health profile of older versus younger girls in terms of violence, nutrition and school attendance, suggesting the need for more nuanced intervention approaches. We found that although the majority of girls attend school, absences were common and many dropped out of school as they got older. Younger and older girls faced violence, especially emotional and physical violence perpetrated by parents and other family members. Stunting affected almost half of girls and many had low BMIs. Although the percentage of early childbearing was relatively low in this setting, the majority of older girls had not heard about contraception, implying that more accessible forms of information on sexual health and family planning are necessary.

Our study benefits from a large community-based sample of girls from largely tribal communities, but has some limitations. Most of the girls were from Ho tribe communities which may limit the generalisability of the study results to girls from other tribal communities. A minority of girls stayed in hostels outside the study villages and could not be included in the study. We were unable to identify a concise and locally validated tool to comprehensively assess mental health. We therefore used the BPC, which has good psychometric properties. We are planning to validate the BPM, a replacement for the BPC, in an adolescent population in Jharkhand. Compared to previous surveys in Jharkhand, we identified a relatively low prevalence of early marriage [28]. Ho communities, the most common tribal group in the study area, may encourage relatively delayed marriage compared to other groups in Jharkhand. The discrepancy could also be due to recent targeted programmes and campaigns (http://www.oxfamindia.org/blog/542/singing-their-way-out-child-marriage)[29, 30], an increasing number of girls staying in school [28], and families knowing the legal age of marriage and being reluctant to identify underage married girls in their household.

The prevalence of stunting in our sample was relatively high compared to a previous estimate of 33.2% among girls aged 15–19 in India [31]. This suggests a large burden of chronic undernutrition in rural Jharkhand, which may have adverse effects on adolescent mental development and educational attainment, with potential long term consequences for employment and earning capacity [32].

The prevalence of thinness (below − 2 SD BMI for age) in our study was higher in younger girls (14%) compared to older girls (6.4%), though lower overall compared to previous surveys. India’s Clinical, Anthropometric and Biochemical (CAB) 2014 Survey reported a prevalence of 28% thinness among girls aged 5–18 years living in rural Jharkhand, and 19% in rural West Singhbhum district [33]. A study of nutritional status in tribal adolescents aged 10–17 across India reported a higher prevalence of 42% thinness (<5th BMI age percentiles of National Health and Nutrition Examination Survey, NHANES) among girls, however these data were collected in 1998–99 [34].

We were unable to clinically assess anaemia, though we would expect the prevalence to be high based on findings from the CAB survey suggesting a prevalence of 86% among girls aged 10–17 years living in rural West Singhbhum [33]. Evidence suggests that iron alone, iron and folic acid, zinc, and multiple micronutrient supplementation can increase serum haemoglobin concentration among adolescent girls [35, 36]. However, in order to identify an appropriate intervention, further investigation into the aetiology of anaemia in this population is warranted. In South Asia, community-based nutrition education and behaviour change interventions have improved girls’ knowledge about nutrition, as well as their dietary practices [36]. Further research is needed to assess the effects of food supplementation and cash transfers on adolescent undernutrition. There is also a need for country and regional data to validate threshold MUAC scores and we hope our data will contribute to this.

Violence is a key health problem facing girls across India. The effects of violence can have both short and long term consequences including injuries, sexually transmitted infections, depression, substance misuse, self-harm, suicide attempts and non-communicable diseases [37–41]. Emotional violence was highly prevalent in our study and included being insulted, humiliated and intimidated. Eve-teasing is a common form of emotional and sexual violence against girls, and includes “staring, stalking, passing comments, and inappropriate physical touch” by boys and men [42]. Consequences for girls may include restrictions on mobility, inability to go to school or work, blame and disrespect from family members [42]. Parents were the most common perpetrators of violence against girls in our study and around a quarter reported witnessing violence between their parents. Community-based interventions that aim to change attitudes and norms that condone violence and gender inequity, and that promote non-violent behaviours could help to reduce violence [43]. Targeting parents as well as adolescents to try to reduce perpetration and exposures in the home may also be beneficial.

Few girls aged 15–19 knew about contraception despite more than a fifth being married. This could reflect the fact that talking about contraception is taboo in the study communities, and girls either had little knowledge on the topic or were reluctant to answer questions about it. A national survey found that young people had limited awareness of most sexual and reproductive health matters [14]. Government strategies to improve adolescent sexual and reproductive health include adolescent friendly health services, community peer-led education sessions and a school-based education programme [44–46]. However, girls may have limited agency to access services and make decisions related to sexual and reproductive health [47]. Future strategies should focus on building adolescents’ self-efficacy as well as improving their sexual and reproductive health knowledge.

Programmes to reduce early marriage and early childbearing often focus on keeping girls in school through conditional cash transfers, school vouchers and other incentives [48]. Our findings suggest that the main reasons for absenteeism, dropping out of school and never attending school are due to employment and domestic work. Research to understand the cost benefits of incentive schemes compared to out-of-school work for adolescents and their families is needed.

What are the implications of our study for future community interventions? India’s national adolescent health and development programme – Rashtriya Kishor Swasthya Karyakram (RKSK) - outlines facility- and community-based interventions to improve adolescent health across a range of areas of health need [46]. In Jharkhand, the programme has not yet reached full coverage, and data on the health of girls in this setting are needed to inform intervention planning and scale-up. Our study identified a wide range of adolescent health needs relevant to RKSK. The programme’s Peer Educator Activity Book, including nutrition, sexual and reproductive health, mental health and violence is therefore appropriate [49]. However, our findings suggest that the content may need to be tailored depending on setting and age and sex of adolescents. For example, overnutrition is a key topic in the Activity Book that currently has limited relevance in rural Jharkhand. Whilst content on alcohol use may be relevant for girls in this setting, activities related to use of tobacco and other recreational drugs may be less useful. Discussions on contraception may be more relevant and acceptable to older girls. Activities to encourage younger girls to stay in school may be beneficial. Formative work to locally adapt the RKSK curriculum is essential, as is implementation research to optimise delivery of the intervention to different groups of adolescents.

## Conclusion

Our study has identified key areas of focus to improve health, nutrition and wellbeing among adolescent girls living in eastern India: reducing violence, early marriage and undernutrition, as well as improving mental health, knowledge about contraception and retention of girls in school. Whilst national government programmes to improve adolescent health broadly cover the main health issues in this population, local assessment of priorities and adaptation of programme content are necessary to ensure programmes are relevant and effective.

## Additional file

Additional file 1: Survey instrument. English language version of survey instrument. (XLSX 27 kb)

## Data Availability

The dataset analysed during the current study is not publicly available, as it constitutes the baseline survey of an ongoing trial. However, it may be available from the corresponding author on reasonable request.

## Abbreviations

BMI: Body Mass Index
BPC: Brief Problem Checklist
BPM: Brief Problem Monitor
FANTA: Food and Nutrition Technical Assistance
ICAST-C: ISPCAN Child Abuse Screening Tools Children’s Version
MGNREGA: Mahatma Gandhi National Rural Employment Guarantee Act
MUAC: Mid-Upper Arm Circumference
NHANES: National Health and Nutrition Examination Survey, NHANES
RKSK: Rashtriya Kishor Swasthya Karyakram
US: United States
